# A Conceptual Model Facilitating the Transition of Involuntary Migrant Families

**DOI:** 10.5402/2011/824209

**Published:** 2011-11-20

**Authors:** Kerstin Linnéa Samarasinghe

**Affiliations:** School of Health and Society, Kristianstad University, 291 88 Kristianstad, Sweden

## Abstract

Refugee families face a complex transition due to the nature of involuntary migration and the process of acculturation. There are several risk factors to the family adaptation process during the transition period, which are sociocontextually environmental dependant. Facilitating a healthy transition for refugee families, therefore, requires the role of nursing to incorporate sociopolitics into the discipline. This paper introduces a sociopolitically oriented and community-driven assessment and intervention model which is based on a family systematic approach. Interventions that aid the families in their acculturation process as well as empowers them to a well-functioning daily life, as per the SARFI model, should be adopted. As such, the future of nursing may provide additional primary health care services for refugee families; this is through a team-led “family nurse” who provides quality care for the family unit in collaboration with other health care professionals and societal authorities.

## 1. Introduction

Transitions in general are concepts of interest to researchers within the field of nursing due to their impact on emotional and physical wellness [1]. As such, effective health promotion should take into consideration transitional factors within the sociocontextual environment that are constraining to family health. Primary Health Care Nurses (PHCNs) who work with migrant families need to focus on these contextual relationships, by gaining an understanding of the external sociocontextual environment in which migrant families live, which in turn are hurdles to achieving a healthy transition. 

The empirical approach to health promotion research should therefore attempt to contextualise the relationships and causal mechanisms that affect the health of the study participants. 

Nursing research can as such further contribute to national as well as global health promotion policies, even taking nursing research into the sociopolitical arena. This contention is supported by D. Whitehead [2] who predicted that health promotion in nursing would advance towards a sociopolitically oriented and community-driven agenda in the future. 

**Figure 1:**
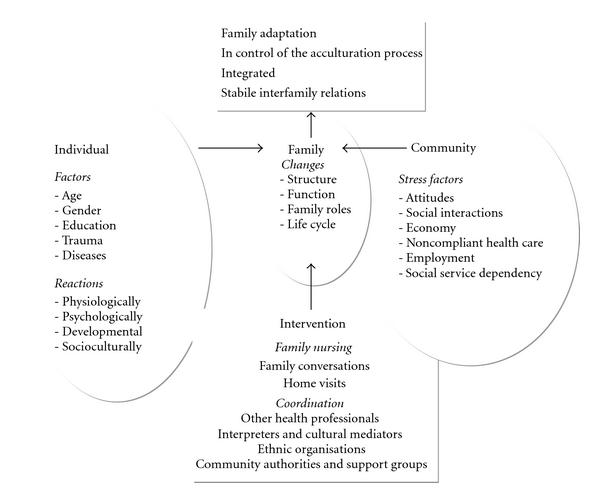
A conceptual model for assessment and intervention of involuntary migrant families in transition; Samarasinghe Refugee Family Intervention Model (the SARFI model) [3, page 49].

The focus of this paper is the Samarasinghe Refugee Family Intervention Model—SARFI (Figure 1). This is a sociopolitically oriented and community-driven assessment and intervention model which enables nurses to uncover external sociocontextual environmental conditions, in turn facilitating the healthy transition for involuntary migrant families. 

The SARFI model was developed by Samarasinghe [3] and is based on a comprehensive understanding of the impact of transition on family health in involuntary migrant families conceptualised by involuntary migrant families [4] and PHCNs [5] in Sweden. 

## 2. Transition Related to Involuntary Migration

Transition originated by moving from one country to another is an accepted stress factor as discussed by Bhugra [6]. According to C. Suárez-Orozo and M. Suárez-Orozco [7] this is further exasperated in cases of involuntary migration due to the multifaceted complexity of an often sudden and unplanned move, commonly coinciding with the loss of home, relatives, friends, and homeland. Additionally, the loss of familiar and culturally determined patterns of behaviour which are part of coherence, guidance, and identity give rise to psychological stress [4]. As such, involuntary migration exposes the family to acculturation, a complex social and psychological process which implies cultural learning and behavioural adaptation of a nonnative country [8]. Families originating from collectivistic-oriented cultures seek refuge in individualistic-oriented cultures that emphasize the autonomy of the individual, risk interpersonal conflicts due to change in the nature of cultural values and family roles of the host country [9, 10].

## 3. The Concept of a “Healthy Transition”

According to A. I. Meleis [11], transition is a combination of change and the individuals' reaction to this change. In the case of involuntary migration, transition is related to the situational change of relocating and the subsequent adaptation to new cultural beliefs through social interactions and the reaction to these changes. Indicators of a “healthy transition” identified by A. I. Meleis et al. [12] are a subjective sense of well-being, mastery of new behaviour, and the wellbeing of interpersonal relationships.

The indicators of a healthy transition in the overall context of migration therefore relate to the *acculturation* process, which refers to physical, biological, economical, political, social, and psychological changes that occur in the learning of a new lifestyle encompassing new values and even proficiency in a new language [13]. The psychological acculturation involves affective, behavioural, and cognitive changes summarized as behavioural shifts involving the adjustment in for instance food, dress, language, and manner of interacting [14]. Acculturation further imposes transformation of identity firstly by encompassing the rejection of the old identity being caught up between the old and the new identity and finally the development of a new identity in turn affecting underlying self-perception processes such as self-esteem and self-efficacy [15]. Moreover, arbitrary interpretations of the behaviour of the refugee by inhabitants of the host country may take place due to the inflicted alexithymia (inability to verbally express feelings) caused by the nonproficiency in the subtlety of the new language [8]. As such, cultural transition may give rise to *acculturative stress* [16], affecting the biochemical system of the body which in turn increases vulnerability to psychological distress and physical disease [17–19]. For a family in cross-cultural transition, changes are likely to occur related to family roles and obligations, memories and communication, relationships with other family members, and changes in family connections with the ethnic community and the host nation state [20]. According to C. E. Sluzki [21] the full impact of acculturation is revealed in full after several years in the new country. Besides, C. E. Sluzki means that family members are frequently unaware of the stressful nature of migration and its cumulative impact on family function. The hallmark of successful acculturation lies in the achievement of a cultural competence encompassing two distinct cultural domains allowing a sense of belonging combined with a successful participation in both [22].

Health in reference to *adaptation* in cases of acculturating families is defined in terms of psychosocial wellness. A definition which is in line with the general definition of WHO: “health is a state of complete physical, mental, and social well-being and not merely the absence of disease and infirmity” [23, page 1]. Moreover, it corroborates with J. Clawson [24] who defined *family adaptation* as a measure of family health. In order to strengthen the resiliency of the family, the positive and negative factors in acculturative stress need to be identified. According to R. L. Punamäki et al. [25] a balance between protective factors, such as education, and traumatizing factors enhances family adaptation. Facilitating* family adaptation* in involuntary migrant families increases resistance against acculturative stress. As such a healthy transition, as indicated by A. I. Meleis et al. [12], which exhibits a sense of control by the individual and family *of their psychosocial health despite past refugee experiences and present acculturation* is a possible outcome. In turn, this supports the stability of interfamily relations whilst acculturating and mastering new behaviours and changes in family roles.

## 4. Involuntary Migrant Families in Cultural Transition in Sweden

Sweden is a receiving country for asylum seeking individuals and families. The country grants permanent residency in accordance with guidelines adopted by the United Nations. Permanent residency is furthermore afforded those seeking to join already approved family members [26]. Sweden represents a culturally pluralistic society with approximately 13% of its 9,4 million population originating from more than 200 countries [27] and of which 46% originate from non-European countries [26]. It is assumed that by the year 2060 approximately 18% of Sweden's population will be foreign born and originating predominantly from non-European countries [27]. The PHCNs are for the most part the involuntary migrant families' first contact with the Swedish health care system [28]. Asylum seekers are offered a free health examination upon arrival, and interpreting services are provided within Primary Health Care (PHC). Once a permanent resident permit is obtained, the family has the same rights to health care as Swedish citizens [28].

### 4.1. Family Stress and Family Function

K. Samarasinghe [3] has described the complexities of relationship between the involuntary migrant families and family adaptation from the perspective of both the migrants and PHCNs [5] encompassing socioeconomic status as well as the experience of various aspects of Swedish society. Findings show that socioeconomical factors such as level of education combined with the process of acculturation as well as host country attitudes impact the outcome of family adaptation. Other factors such as trauma prior to migration, self-perception of identity, and self-efficacy also play a vital role in family interrelationship and consequent family health. 

These studies [4, 5] demonstrate that a* sociopsychosomatic* relationship to family adaptation exists during the cultural transition in Sweden. The principal stressors in terms of family health were found to be *extra-personal stressors *formed within the sociocontextual environment, prolonged uncertainty during the asylum seeking process, unemployment, marginalisation due to dependency on social services, a minimized social network, and the loss of social status resulting from having to accept employment beneath ones level of qualifications. Furthermore, the perception of social authorities' support of children in family conflicts, ethnocentric health care resulting in misunderstandings between family members and health care personnel, feelings of being discriminated against, and xenophobia were also stressors. In addition, living with unprocessed premigration traumas where treatment was unavailable created a stress factor. The specific changes in family life due to the process of acculturation were identified as unclear family roles, changed values in children's responsibility to take care of the elderly, a weakened parental role, and adolescent's rebellious behaviour.

Individuals' reactions to acculturation and the extra personal socioenvironmental stressors were found to manifest several forms of somatic symptoms through the development of physical symptoms such as abdominal pain, chest pain, headaches, muscle aches, gastritis, and eczema and where chronic disorders such as diabetes and asthma were perceived to intensify. Psychological effects such as enuresis in older children, sleeping difficulties, fainting attacks, low self-esteem, depressive and suicidal tendencies together with behavioural changes in the form of aggression, gambling addiction, self-immolation in children, and failure to interact with other children were recorded. Intrapersonal stressors were such as unprocessed trauma causing flashbacks and home sickness, feelings of inadequacy, loss of social status, and even a changed self-perception. Furthermore, developmental health effects were such as difficulties in learning and comprehension of the Swedish language and changes in social behaviour in the form of passivity and criminality. In the existential and spiritual realm effects of hopelessness and disillusion of life were noted. 

Conversely several “protective” factors were recognized such as education, high social standing in the country of origin, and the absence of severe traumatic experiences. After arriving in the new home country, protective factors were accredited to the relative ease of integration and welcoming in to society, socially positive interactions, and a competence affirmation through gaining employment in line with qualifications and with similar status as employment previously held in the country of origin. Independence from social welfare handouts and having clear roles within the family were further regarded to contribute to family health [3].

### 4.2. Critical Events during Transition

According to A. I. Meleis et al. [12] most transition experiences involve *critical points or events*. The asylum seeking period was identified as such, affecting the well-being of the whole family by contributing to the weakening of the parental role [3]. According to D. L. Sam [14] this period signifies a high risk of acculturative stress which could increase symptoms of trauma. The reduced family cohesion during this particular period is attributed to the indeterminate time of living in uncertainty [3]. The conclusion is reinforced by G. Roth [29] who exemplified that Kosovo Albanian refugees' mental health deteriorated due to the prolonged waiting in Sweden for the obtaining of asylum. The period following the granting of a Permanent Residence Visa was further recognized as critical due to postasylum stress reactions manifested as depression and physical diseases, such as eczema, gastritis, and body pain [3]. Physical and mental reactions related to prolonged stress in refugees are universally acknowledged [30]. Family reunification was yet another critical event [3] due to the increased risk of family conflicts caused by the varying degrees of acculturation within the family. This conclusion supports findings on reunification of Congolese family members in Canada [31]. 

## 5. A Conceptual Model for Facilitating a Healthy Transition for Involuntary Migrant Families

The SARFI model was developed as a tool for nurses within Primary Health Care to assist in the assessment and intervention in family adaptation of involuntary migrant families in cultural transition based on the following objectives:

- to manage the acculturation process,
- to integrate the family into society,
- to achieve stable family relationships.

The model will enable nurses to facilitate adaptation for involuntary migrant families in relation to the previously identified hallmarks of a “healthy” transition [12] by identifying stress factors threatening the wellness of the family as well as protective factors contributing to family wellness. This can be done through the assessment of facilitating vis-à-vis constraining factors to adaptation from an individual, family, and community perspectives. Additional variables such as lifecycle, gender, and age have been incorporated into the model in conjunction with physiological life cycle changes in order to extend the depth of assessment of acculturative stress [32]. 

The SARFI model is based on supportive conversations on transition experiences from a family perspective. Since acculturative stress in one family member can create stress in another member of the family [33], each individual in crisis is, as such, a potential risk factor to the wellness of the family as a whole. The model has sought inspiration from the Calgary family-focused care model developed for nurses by L. M. Wright and M. Leahey [34]. The foundation of the Calgary-based family care model is the enabling of the nurse to construct an environment for change based on the reflections of the family's core beliefs about the prevailing illness of a family member. As insight is a prerequisite for understanding, it is fundamental to *normalizing* effects caused by stressors that the involuntary migrant families gain insight into stress factors that influence interfamily relationships through the cultural transition. That the families receive support to identify and reflect upon stressors which instigate structural changes within the family unit in turn risking interfamily conflicts is the essence of the SARFI model. Moreover, it assists in the counteraction and coping strategies to these changes. 

### 5.1. Health Supportive Family Conversations

The conversations with one or several family members can advantageously be conducted within the family home, as home visits have shown to be a valuable nursing tool as it allows for an increased dept of understanding of the family [35]. The PHCNs' role is to congregate the family and assist it in reflecting upon acculturation changes. Interpreters and cultural mediators can provide key supportive roles in the process when the family is as yet not proficient in the new language. 

The *first conversations* with the family should focus on the family's perception of the differences in cultural patterns and behaviour between their country of origin and the new home country. This could help illustrate their core beliefs in regard to cultural norms. It would support the reflection of ideas surrounding cultural and individual views on topics such as sickness and health, gender roles, child rearing, communication, and behavioural roles within family and society to name a few. Questions can be shaped by the PHCN in conjunction with the family to better capture the particularities of the family and their culture and situation. 

Similarly, the PHCN should reflect and discuss prevailing norms and behavioural patterns within the social framework of the new home country. Information regarding the organisation of society, how health care is provided including health laws and statues are of relevance. The PHCN should function as a support and information source to the family in discovering the existing and perceived cultural differences and encourage the family and its members to reflect on their views and how these are similar to or different from the prevailing norms. This is beneficial so far as it allows the family to understand the sociocultural context within which they now exist and acculturate. Moreover, it supports the family in reflecting on thoughts around the role of identity and how the acculturation process impacts this, individually as well as a family.

In the *second set of family conversations* the PHCN should discuss topics concerning the daily life in the family's country of origin. These can then be used as a back drop for the family to reflect on their expectations of the new society and the ways in which these could cause structural changes within the family unit depending on various family members' thoughts and the reality available to the family in meeting these expectations. The ability to “talk about ones life and experiences” can be helpful in enabling trust and confidence to grow between the family and the PHCN. Moreover, open questions inviting narratives of past experiences can function as a tool to process and confirm the role of identity within the family. 

In *the third round of conversations*, the experiences of specific issues related to cultural differences are discussed. These are then collectively analysed as a way for the family to reflect on how these may or could impact the family's structure and function. A useful instrument to illustrate family relations is the three-generational genogram. It illuminates an overview of family relations how these have been affected [34]. How have the families' roles changed from the previous home country and the present one? Have family functions changed in relation to society and state? Have gender roles changed? Have these changes affected the family's finances and family cohesion? Have the family experienced any culture shocks and misunderstandings, and, if so, what impact has this had on their daily lives? Questions can be developed by the PHCN and discussed within context of the family's physiology, psychology, socio-cultural, and existential wellness.

The use of an *ecomap* will help illustrate the structure, function, and developmental life cycle of the family as well as their social network [34]. It can further help in depicting interaction patterns within the family and between families and the community. Both the genogram and ecomap can aid the PHCN in clarifying acculturation to the family and the possible stressors which can accompany the process. 

In *the fourth set of conversations* family patters around cultural amalgamation are brought up for reflection. Gaining an awareness of these patters and their subsequent effects can assist family members' understanding of the particulars of each other's acculturation process. This in turn permits discussion around optimal ways to manage and adapt in order to promote wellness within the family.

### 5.2. When Should Family Conversations Start?

The starting point and time frame of the conversations are to be qualitatively assessed by the PHCN and viewed in relation to each family and their particular situation and any critical points that they are experiencing. For instance, the asylum seeking period is regarded as a critical point, and it has been recorded that parental roles are weakened during this time as stressors constrain their previous parental function [5]. The PHCN may as such decide to commence conversations during this time focusing on conversations strengthening parental roles.

### 5.3. How to Promote Integration?

The PHCN can be beneficial to the families' process of integration by cooperating with ethnic organisations, cultural mediators, and other professionals within the area of health care. Additionally promoting the collaboration between municipal and community-based network organisations and the family has been found to be beneficial to the integration process [3]. 

In short, in encounters with involuntary immigrants nurses should adopt a holistic approach to wellness. This conclusion corroborates with K. Samarasinghe et al. [36] who showed that PHCNs having such an approach in their promotion of involuntary migrant families' health empowered the family to function well in everyday life.

## 6. The Future of Nursing: Implications for Nursing Practice and Research

Migrant health needs are largely managed within the Primary Health Care sector, and public health nurses or community health nurses are therefore ideal health providers [37]. To enhance family wellness and family cohesion, PHCNs should act as mediums in facilitating involuntary migrant families' cultural transition by empowering the family to be in control of the process of acculturation. As such, all dimensions of health pertaining to individuals as well as to the family unit should be incorporated in the promotion of health. The current environmental paradigm of nursing is however psychosocial and client orientated and does not adequately explain problems originating from the contextual environment [38]. In cases of involuntary migrants nurses may therefore provide additional primary health care services through a “family nurse” who can provide care for the family unit in coordination with other health care professionals and societal authorities. In adopting a family system perspective when conducting conversations about transition experience Primary Health Care Nursing is enabled in improving the level of refugee families. As such, sociocontextual environmental stressors are exposed, allowing for the addressing of these on an individual, family, and community levels. 

Further research on the topic of health and involuntary migration would benefit from the emphasis of the essential nature of healthy transitions and the ways in which these can be facilitated by engaging refugee families in participatory research. Participatory action research (PAR) is increasingly recognized as a viable approach to address complex public health problems as it seeks to democratize research by including all stakeholders, from informants all the way to the principal investigator, in all research activities [39]. Combining participatory action research methods with health promotion activities therefore empowers the future adaptation and wellness of refugee and immigrant families.

## 7. Conclusion

Involuntary migration and the consequent cultural transition are known to challenge family wellness. Facilitating involuntary migrant families' transition which aims for a *successful adaptation* to a new sociocultural environment requires an expanded approach from the nursing practice. It will require from nurses the ability to work with a sociopolitical framework and relating this to the objective of family wellness. A “family nurse” utilizing the sociopolitically oriented and community-driven SARFI intervention model in working with involuntary migrant health is proposed. Further research using participatory action research approach is also recommended. Promoting wellness for the involuntary migrant families will be a challenge for nursing in the future.
